# Compartmentalization and Cell Division through Molecular Discreteness and Crowding in a Catalytic Reaction Network

**DOI:** 10.3390/life4040586

**Published:** 2014-10-29

**Authors:** Atsushi Kamimura, Kunihiko Kaneko

**Affiliations:** Department of Basic Science, The University of Tokyo, 3-8-1, Komaba, Meguro-ku, Tokyo 153-8902, Japan; E-Mail: kaneko@complex.c.u-tokyo.ac.jp

**Keywords:** protocells, compartment, catalytic reactions, minority control, crowding

## Abstract

Explanation of the emergence of primitive cellular structures from a set of chemical reactions is necessary to unveil the origin of life and to experimentally synthesize protocells. By simulating a cellular automaton model with a two-species hypercycle, we demonstrate the reproduction of a localized cluster; that is, a protocell with a growth-division process emerges when the replication and degradation speeds of one species are respectively slower than those of the other species, because of overcrowding of molecules as a natural outcome of the replication. The protocell exhibits synchrony between its division process and replication of the minority molecule. We discuss the effects of the crowding molecule on the formation of primitive structures. The generality of this result is demonstrated through the extension of our model to a hypercycle with three molecular species, where a localized layered structure of molecules continues to divide, triggered by the replication of a minority molecule at the center.

## 1. Introduction

Understanding how cellular basic components are integrated into a reproducing cell is essential to unveil the origin of life, to give a comprehensive insight into cellular life, and to experimentally synthesize an artificial cell from its basic elements [1]. Several theoretical studies linked a set of catalytic reactions with a reproducing cell [2,3,4,5,6]. In particular, a hypercycle model, in which different molecular species mutually catalyze the replication of each other, was proposed to give stable replications of polymers carrying information [7,8]. Further, compartmentalization of the catalytic molecules enhance the robustness of the growth against parasites [9,10,11,12].

With the stable polymer replication, we may ask rather naive questions. In the present cell, a specific molecule (DNA) that carries “genetic information” is separated from molecules carrying metabolic reactions. Is such separation a necessary course for a system with reproduction and evolvability? Is there a general mechanism for such separation in a reproducing reaction network system, even if we do not assume material properties as in DNA or RNA? These questions are related to a mechanism of “genetic takeover”, originally posed by Cairns-Smith [13] and later by Dyson [2].

To answer these questions, a hypothesis has been proposed: a minority molecular species in the hypercycle tends to play the role of heredity-carrier, which also provides evolvability [14]. As a first step, the simplest hypercycle is considered: two molecular species (*X* and *Y* ) mutually catalyze the replication of each other. They are encapsulated in a compartment, and the compartment (protocells) undergoes divisions when the number of molecules inside exceeds a given threshold. When one of the species, *Y* , is assumed to have a slower replication speed than that of *X*, the system reaches a state where, in each compartment, there are only a few *Y* and large population of *X*. The few *Y* molecules were shown to control the behavior of the protocell: that is, a small change in the minority species results in a relatively stronger influence on replication of the other molecular species. In this hypothesis, the existence and division of a compartment that supports catalytic reactions are pre-assumed.

One of the important remaining questions regarding the origin of life is at which stage did cellular compartmentalization takes place and how all the molecules are incorporated into a closed, compartmentalized structure. To gain insight into this question, it is important to develop a simple mechanism connecting compartmentalization with the evolution of genetic molecules. Also, in the synthesis of an artificial cell, it is important to understand how to synchronize division of a cell with replication of molecules inside, particularly, with respect to DNA [15,16].

Discreteness [17,18,19,20] and crowding [21] of molecules have been suggested as two important aspects of biochemical reactions in a cell but have been neglected in conventional reaction-diffusion equations. The discreteness of molecules focuses on the very small number of specific molecules in the cell, and the crowding of molecules emphasizes the high concentrations of molecules with large exclusion volumes in the cellular environment.

Recently, we have considered a hypercycle with two mutually catalyzing chemicals to demonstrate that the reproduction of a protocell with a growth-division process emerges when the replication and degradation rates of one chemical are slower than those of the other, and replication causes molecular crowding [22]. In addition, the protocell divides after the minority molecule is replicated at a slow synthesis rate. Thus, synchrony between cellular divisions and molecular replications is achieved. While the model studied in [22] deals with Langevin dynamics in a continuous space, studying a lattice model will also help clarify the effect of exclusion volumes.

In this paper, we simulate a cellular automaton model with two mutually catalyzing chemicals to show that the synergetic effect of discreteness and molecular crowding is essential to give rise to the primitive cellular structure. In addition, we show the generality of the results by extending our model to a hypercycle with three molecular species.

## 2. Model

We consider a two-dimensional square lattice *L_x_* × *L_y_* with periodic boundary conditions. Each site is empty or occupied by no more than one molecule to ensure appropriate representation of the exclusion volume of molecules. The species identity is represented by a “color” of the molecule, and replications of molecules occur based on the catalytic relationship between the species as described below. We adopt discrete simulation step, and update the system at each step by three processes: replication, degradation and diffusion. Within each of the processes, the following procedure is applied to every molecule in a random order.

**Figure 1:**
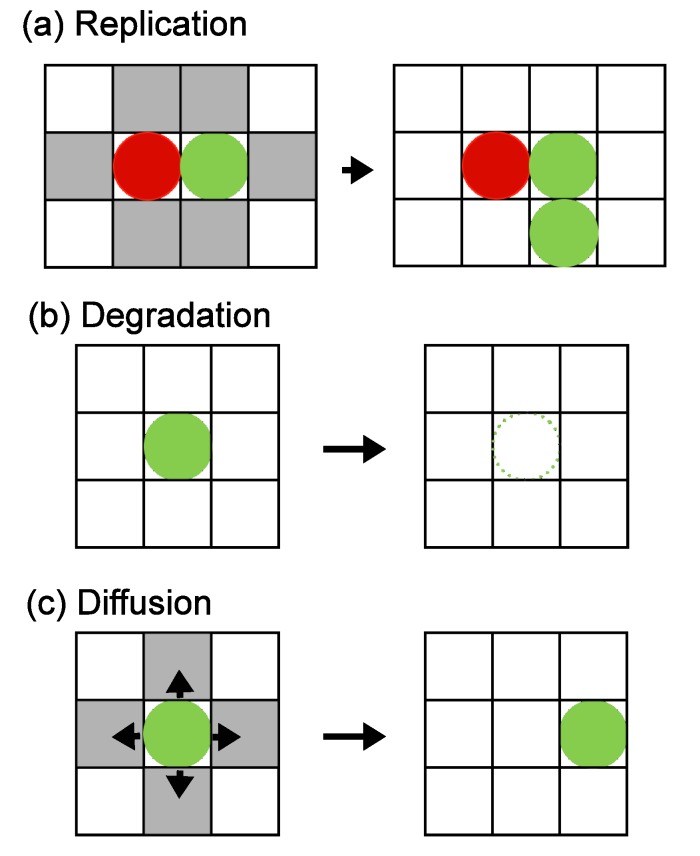
Three update processes of our model. The left column shows the configuration before each process, and the right column displays the outcome of the process. (**a**) Replication: If two catalytically related molecules are located next to each other, replication can occur, and the product molecule is added to one of the six neighboring sites (gray) if an empty site is available. (**b**) Degradation: In each step, each molecule is removed from the system with a fixed probability. (**c**) Diffusion: In each step, every molecule moves to one of the four nearest neighboring sites (gray) if a site is empty.

The first process is molecular replication, as outlined in Figure 1a. When a molecule is located in a site neighboring its catalyzing molecule, replication occurs with a given probability corresponding to the replication rate, and a new molecule is added at a randomly chosen empty site among the six sites neighboring the reaction pair. The example in Figure 1a illustrates a case in which the replication of a green molecule is catalyzed by the red molecule. If all six neighboring sites are occupied, the new molecule is not replicated.

The second process is molecular degradation. In each step, degradation occurs by removing each molecule with a given probability representing the degradation rate of the system (Figure 1b).

The third process is diffusion. In each step, every molecule moves to one of the four nearest neighboring sites if the destination site is empty (Figure 1c). Hence the time steps and lattice sizes are chosen so that a random walk occurs at each step. The destination site is chosen randomly from the four sites, or the molecule remains at the original site if all four sites are occupied.

## 3. Simulation Results

### 3.1. Two Mutually Catalyzing Molecules

The simplest hypercycle is the case of two molecular species *X* and *Y* that mutually catalyze the replications as follows:

$$X+Y\overset{p\gamma_{X}}{\to }2X+Y,\;\;X+Y\;\overset{p\gamma_{Y}}{\to }2Y+X.$$

When *X* and *Y* are located next to each other, replication can occur with probability *p*, as explained in Section 2. If an empty site is available, a new molecule is added, and the molecular species is assigned as *X* or *Y* , with probabilities *γ_X_* and *γ_Y_* = 1 *−* *γ_X_*, respectively. Here we denote *p_Y_* = *pγ_Y_* and *p_X_* = *pγ_X _*. Degradations occur as *X* → 0 or *Y* → 0 with degradation probabilities *a_X_* and *a_Y_*, respectively. 

Simulations are carried out from the initial condition in which a single *Y* is located in a group of *X* with dimensions *L*_ini_ × *L*_ini_. Here, the value of *L*_ini_ is fixed to 10; however, the exact dimensions are not important if they are sufficiently large.

**Figure 2:**
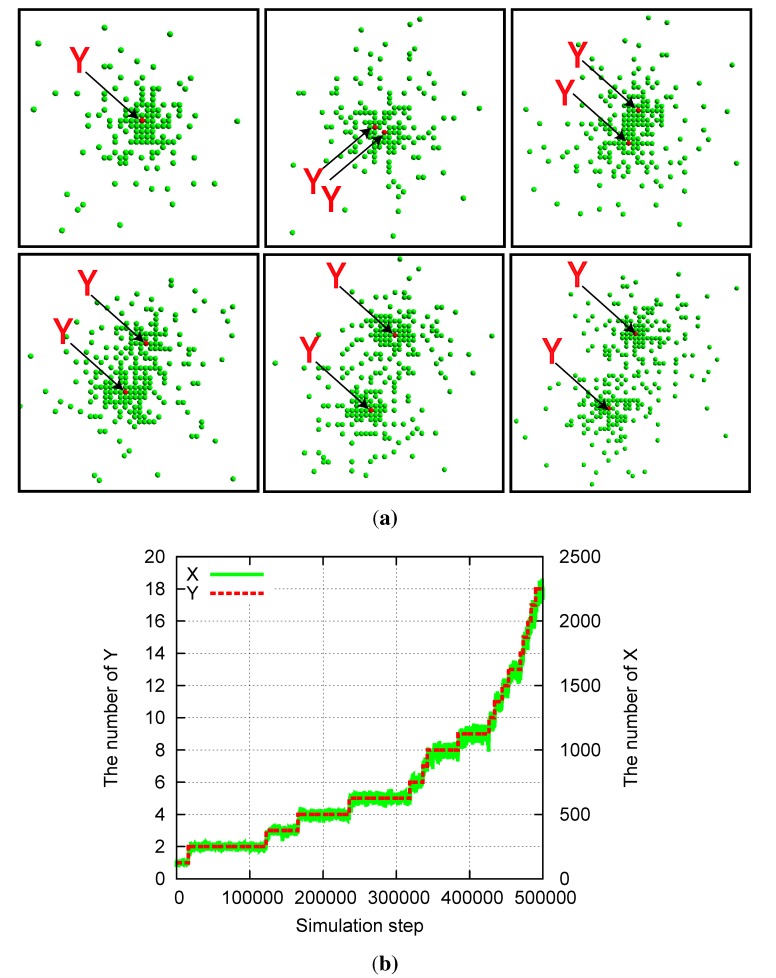
Division of a compartmentalized structure for the model of two mutually catalytic molecules *X* (green) and *Y* (red). Parameters are *p_Y_* = *pγ_Y_* = 5 × 10^−^^6^, *p_X_* = 1 *−* *p_Y_*, *a_X_* = 0.01, *a_Y_* = 0, and *L_x_* = *L_y_* = 1000. (**a**) Snapshots of the system. *Y* molecules are highlighted by arrows. The time steps increase from top left to top right, and then from bottom left to bottom right. Snapshots are shown for every 500 steps from 25,000 to 27,500. (**b**) Time evolution of the number of *X* and *Y* molecules corresponding to the data of (a).

When the replication and degradation rates for *Y* are considerably slower than those for *X*, the mutually catalytic replication reactions result in the emergence of a localized spatial structure. This agrees with the structure reported in [22], in which a single *Y* is surrounded by a group of *X* (Figure 2a).

In addition, division of the structure is observed synchronously with the replication of *Y* . As *Y* replicates at a slow rate, the two *Y* molecules start to diffuse apart (Figure 2a). As each *Y* catalyzes the replications of *X*, several replicated *X* molecules crowd around each of *Y* . The size of each structure is determined by the average distance over which *X* molecules diffuse before they degrade. Then, when *a_Y_* is much smaller than *a_X_*, the two *Y* molecules diffuse over a larger distance than the size of the cluster, forming a dumbbell-like cluster around the *Y* molecules and resulting in division of the cluster. Through division after the replication of *Y* , the number of *X* molecules also doubles (Figure 2b), and recursive growth by successive replications of *Y* molecules is possible. Here, we also note that this lattice model allows for a case in which the distance between the two *Y* molecules decreases again through diffusion, leading to a decrease in the number of *X* molecules as the effective replication rate of *X* decreases.

The division process is enabled by the synergetic effect of molecular discreteness and crowding. Shnerb *et al.* [17,18] investigated a system with a reaction *X* + *Y* → 2*X* + *Y* and showed that the discrete number of *Y* molecules leads to localized subpopulations and results in a proliferating phase in which the number of *X* molecules approaches infinity, even for parameter ranges in which the corresponding reaction-diffusion equation leads to extinction of *X*.

In our model, the realistic molecular exclusion volume effect introduces a feedback effect in which crowded *X* molecules suppress molecular replications because at most four *X* molecules can react with the single *Y* in each step and the crowded molecules result in a lack of space near the reacting pair. Thus, a localized structure is formed as a “quasi-stationary” state, in which the number of *X* molecules fluctuates around an average value unless *Y* replicates or degrades. As *Y* replicates, the number of *X* molecules shows a step-like increase (Figure 2b).

**Figure 3:**
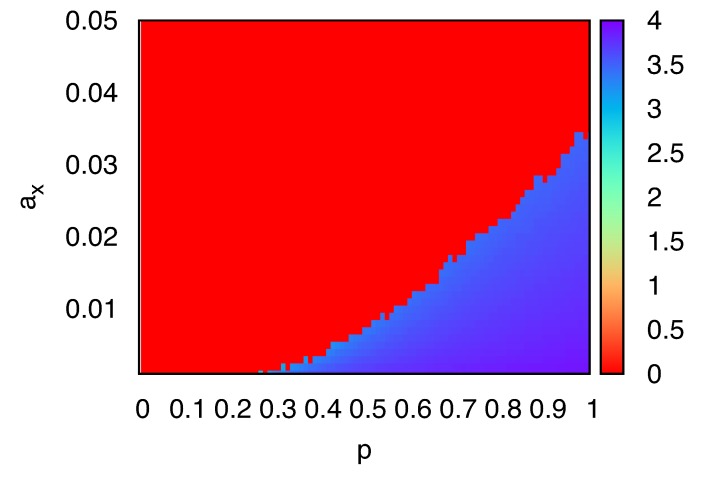
The average number of *X*, *n_X_*, in the four nearest-neighboring sites around a single *Y* molecule, plotted as a function of *p* and *a_X_*. Here, *γ_Y_* = *a_Y_* = 0.

To further clarify the impact of molecular crowding, we investigated the density of *X* molecules in the sites neighboring the single *Y*. The average number of *X*, *n_X_*, in the four nearest-neighboring sites of the single *Y* is shown in Figure 3, as a function of *p* and *a_X_* corrected for the change in number of *Y* molecules, *i.e.*, replication/degradation of *Y* are absent in computation of the average crowdedness. There are two distinct planes *n_X_* = 0 and *n_X_* *>* 3.5, against the parameters *p* and *a_X _*. In the region *n_X_* = 0, no replication occurs and degradation of *X* leads to extinction. In the region *n_X_* *>* 3.5, localized structure appears. We note that, in simulations with parameters near the boundary between the two regions, the behavior of the system depends on samples: the localized stationary structure or extinction. As long as the system maintains the structure, the value of *n_X_* is approximately the fully-occupied value 4. These results suggest that the discreteness of *Y* molecules and the exclusion volume effect set an upper limit for the effective replication rate of *X*. If the degradation rate of *X* is smaller than the replication rate, replication of *X* results in the crowding of *X* around the single *Y* . If the degradation rate of *X* is greater than the replication rate, extinction of *X* is observed.

**Figure 4:**
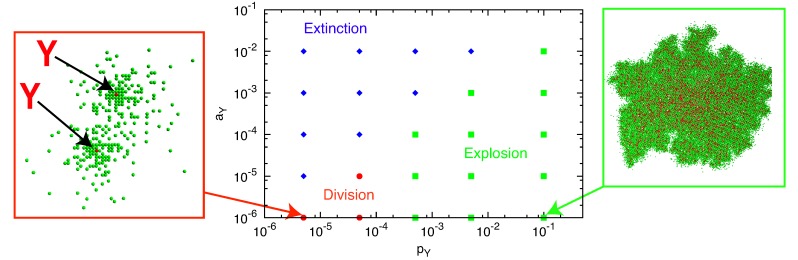
Three types of behavior from the initial condition in the *p_Y_*-*a_Y_* space. Here, *p_Y_* = *pγ_Y_* is changed by *γ_Y_* *<* 0.5 with *p* fixed to one. *p_X_* = 1 *−* *p_Y_* and *a_X_* = 0.01. The plane is divided into three regions: extinction (blue diamonds), division (red circles) and explosion (green squares). Typical snapshots are also shown for the division and explosion regions. In the left snapshot, the two existing *Y* molecules (red) are highlighted by arrows.

Depending on the parameters *a_X_*, *p_X_*, *a_Y_* and *p_Y_*, we observe three types of behavior for the *p_Y_*-*a_Y_* plane: extinction, division and explosion (Figure 4). The division process is observed for smaller *p_Y_* and *a_Y_* in the region of Figure 4 designated by red circles. For larger *p_Y_* (explosion region; green squares), both molecules grow and maintain a single cluster with mixed configuration (Figure 4, right). For larger *a_Y_* (extinction; blue diamonds), *Y* degradation is followed by the degradation of all *X* molecules, resulting in the extinction of all molecules.

### 3.2. Three Cyclically Catalyzing Molecules

After the two-species system, the next simplest case is a hypercycle consisting of three molecular species (*X*, *Y* and *Z*) that catalyze the replications of other species cyclically as follows:

*X* + *Y* → 2*X* + *Y , Y* + *Z* → 2*Y* + *Z*, *Z* + *X* → 2*Z* + *X*.

Here, we denote the replication probabilities of *X*, *Y* , and *Z* by *p_X_*, *p_Y_*, and *p_Z_*, respectively. Degradations also occur as *X* → 0, *Y* → 0 and *Z* → 0 with degradation probabilities *a_X_*, *a_Y_* and *a_Z_*, respectively.

Simulations are carried out from the initial condition in which a single *Y* is located in a box with dimensions *L*_ini_ × *L*_ini_ with randomly located *X* and *Z* molecules.

**Figure 5:**
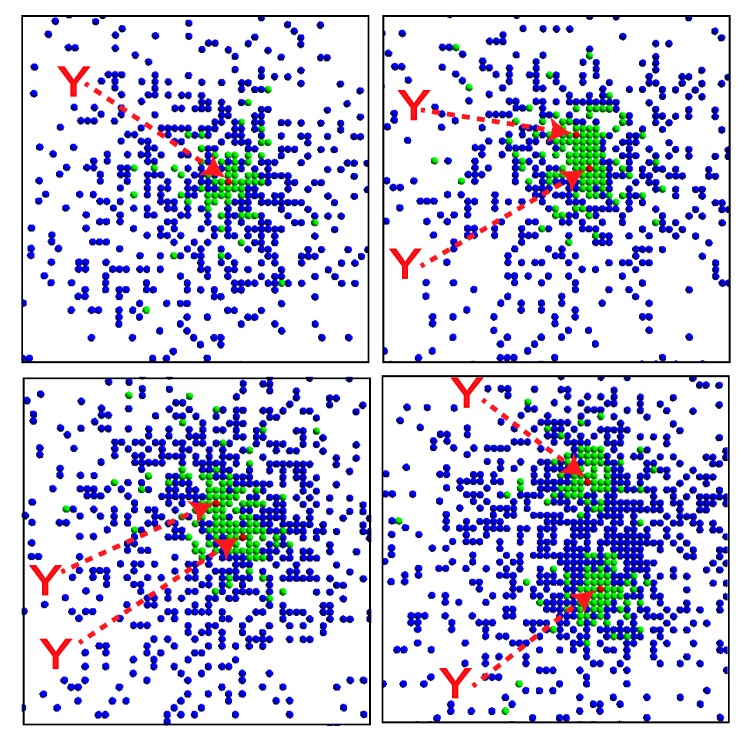
Snapshots of the system. The *X*, *Y* and *Z* molecules are respectively denoted by green, red and blue dots. The *Y* molecules are highlighted with arrows. The time steps increase from top left to top right, and then from bottom left to bottom right. Snapshots for every 1000 steps are shown.

**Figure 6:**
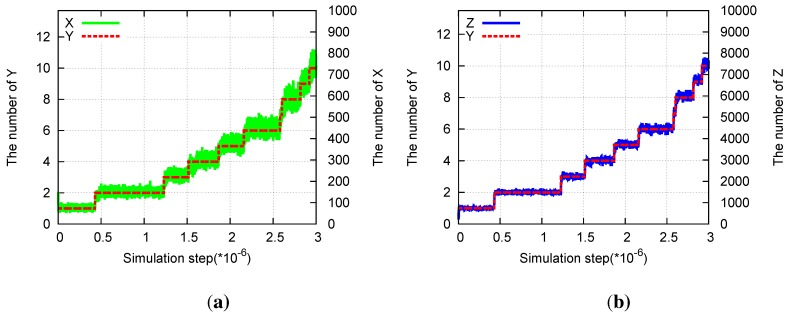
Division in the three-species hypercycle. Parameters are *p_X_* = 1, *p_Y_* = 0.0001, *p_Z_* = 0.01, *a_X_* = 0.01, *a_Y_* = 0, *a_Z_* = 0.001. (**a**) Time evolution of the number of *X*(green) and of *Y* (red) molecules. (**b**) Time evolution of the number of *Z*(blue) and of *Y* (red) molecules.

For a specific set of parameters, the system organizes a localized nested structure in which a single *Y* is surrounded by several *X* molecules, which are surrounded by a number of *Z* molecules (Figure 5, top left panel). When the replication rate of *Y* is small, a cluster of *X* molecules is distributed around *Y* because *Y* catalyzes the replication of *X*. *X*, in turn, catalyzes the replication of *Z* such that several *Z* molecules are distributed around the cluster of *X* molecules. Replication of *Y* is catalyzed by *Z*, which is generally separated from the *Y* molecule. When a *Z* molecule diffuses into the cluster of *X* molecules surrounding *Y* , a new *Y* molecule can be replicated. After replication, the two *Y* molecules diffuse apart, and the structure gradually divides (Figure 5). The replication of *Y* also doubles the numbers of *X* and *Z* molecules, and recursive growth by successive replications of *Y* is possible (Figure 6).

**Figure 7:**
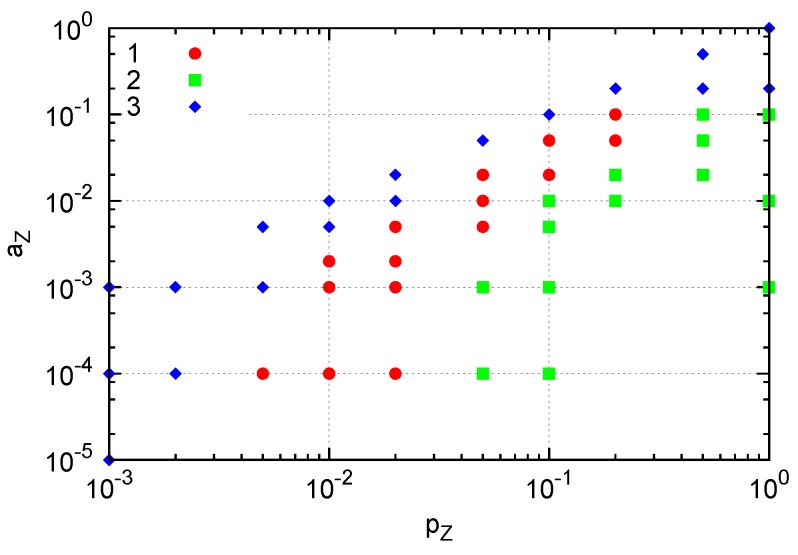
Three types of generated structures. A single *Y* molecule is fixed by suppressing changes in *Y* molecule (i.e., *p_Y_* = *a_Y_* = 0). (1) A cluster of *Z* molecules surrounding a cluster of *X* molecules around a single *Y* (red). (2) Extinction of both *X* and *Z*: crowding of *Z* results in extinction of *X*, and subsequent extinction of *Z*. (3) Extinction of *Z* occurs. Here, *p_X_* = 1, *p_Y_* = 0, *a_X_* = 0.01 and *a_Y_* = 0.

In our model, new molecules are replicated when an empty site is available near the reacting pair. Hence the effective replication rate depends on the spatial distribution of molecules: *i.e.*, replications are suppressed in crowded situations. With three species, a pair of reacting species is more difficult to interact than in the two-species case. Still, a localized cluster sustaining replication is formed in a certain range of parameters. The parameter region of *p_Z_* and *a_Z_* in which the localized structure appears when a single *Y* molecule is fixed is indicated by red circles in Figure 7. For smaller *p_Z_* or greater *a_Z_* values (blue diamonds), extinction of *Z* occurs and prevents the growth of the structure. On the other hand, greater *p_Z_* values (green squares) yield crowded *Z* molecules, effectively preventing replication of *X* and resulting in the extinction of *X* and subsequently extinction of *Z*. This result indicates that formation of a dividing cluster crucially depends on the relative replication and degradation rates between *Y* and the other species as in the two-species case, but also between *X* and *Z* molecules in this three-species case. In the parameter space of Figure 7, for example, *p*_*Z*_ should be smaller than *p*_*X*_ for the emergence of the cluster.

## 4. Conclusions

Herein, we demonstrated that a primitive localized cluster appears in a cellular automaton model, with mutually catalytic replicating reactions and consideration of molecular exclusion volumes when the replication and degradation rates of one molecular species are slower than those of the other. Both the lattice model with cellular automaton dynamics studied herein and the continuous space model with Langevin dynamics studied in [22] demonstrate that the synergetic effect of molecular discreteness and crowding leads to the division of a compartmental structure.

In addition, we show in the lattice model that a nested structure appears when there is a minority species in a hypercycle consisting of three molecular species, and the structure divides with the replication of the minority species.

Throughout biological systems, polymer replication is an essential process. Such macromolecular polymers consist of many units and are commonly very large. Thus, as replications progress, the excluded volume for such macromolecules is significant and crowding is inevitable. In our study, molecular crowding is a natural consequence of the replication processes, without which the population of molecules goes extinct. On the other hand, achievement of a high local concentration is advantageous for further growth, compared with a dilute solution case, as reactions can occur more frequently. Such sustained crowdedness is also relevant to the development of a more complex cellular structure.

In addition to the replication of polymers, the reproduction of a compartmentalized cell-like structure is important in understanding the origin of life. When synthesizing protocells, it is important to synchronize cellular division with the replication of the molecules inside, particularly, that of DNA [15,16]. Our model exhibits autonomous synchronization between the replication of the information (minority) molecule and the division of a cell-like structure, thereby providing a foundation for future experiments to develop a mechanism of robust compartmentalization by introducing membrane molecules into our model. Spontaneous crowding is physically realistic and ubiquitous for proteins and nucleic acids encapsulated by lipid vesicles and has been incorporated in recent experiments [23]. Even under such conditions, the present mechanism considering a minority molecule and exclusion volume naturally gives rise to compartmentalization and synchronous division with a replication of the specific molecule; otherwise, molecules become extinct or the system becomes a mixture of excessively crowded molecules.

In the previous study [14], which pre-assumed a compartment and its division supportive of catalytic reactions, the growth rate of a protocell with a minority molecule is generally slower than for a protocell in which all members (*X* and *Y*) are equally abundant. The growth speed generally indicates the fitness according to Darwinian evolution, and how a system with a minority molecule can evolutionarily triumph remains unclear. Under the crowded conditions caused by the replication of polymers, such evolution may not be dominated by growth speed because the condition will slow down the uptake of chemical resources for polymer synthesis, and their availability and variability are essential for growth. In such a case, the coexistence of diverse protocells with different compositions is possible [24], and protocells with a minority molecule will be able to survive and more likely to evolve.

Theoretical concepts, albeit drawn from simple setups, can sometimes provide a novel principle on what life is, as seen in the classic studies of Turing’s pattern formation [25] and Eigen’s error catastrophe and hypercycle [7,8]. Although the model discussed in the present paper is simple and abstract, theoretical conclusion drawn from the study is general and can also provide a novel guide to design a constructive experiment for artificial cells as well. Indeed, the present theory sheds new light on the synchronization of replication of information molecule and division of a cell, while it suggests a way to design an artificial, reproducing cell even without explicit synthesis of a membrane.
